# Placental malaria and low birth weight in pregnant women living in a rural area of Burkina Faso following the use of three preventive treatment regimens

**DOI:** 10.1186/1475-2875-8-224

**Published:** 2009-10-07

**Authors:** Alfred B Tiono, Alphonse Ouedraogo, Edith C Bougouma, Amidou Diarra, Amadou T Konaté, Issa Nébié, Sodiomon B Sirima

**Affiliations:** 1 Centre National de Recherche et de Formation sur le Paludisme, Ministère de la Santé, Ouagadougou, Burkina Faso; 2 Groupe de Recherche et d'Action en Santé (GRAS), Ouagadougou, Burkina Faso

## Abstract

**Background:**

The weekly chemoprophylaxis of malaria during pregnancy with chloroquine (CQ) has become problematic with the increasing resistance of *Plasmodium falciparum *to this drug. There was a need to test the benefits of new strategies over the classical chemoprophylaxis. This study was conducted to provide data to the National Malarial Control Programme for an evidence-based policy change decision making process. It compares the efficacy of two IPT regimens, using chloroquine (CQ) or sulphadoxine/pyrimethamine (SP), with the classical chemoprophylaxis regimen using CQ in reducing the adverse outcomes of malaria infection, for the mother and the foetus.

**Methods:**

Pregnant women attending the first antenatal care visit were randomly assigned to one of the three treatment regimens. They were subsequently followed up till delivery. Maternal, placental and cord blood samples were obtained upon delivery to check for *P. falciparum *infection.

**Results:**

A total of 648 pregnant women were enrolled in the study. Delivery outcome were available for 423 of them. Peripheral maternal *P. falciparum *infection at delivery was found in 25.8% of the women. The proportion of women with maternal infection was significantly lower in the IPTp/SP group than in the CQ group (P << 0.000). The prevalence of placental malaria was 18.8% in the CWC/CQ group; 15.9% in the IPTp/CQ group and 10.6% in the IPTp/SP group. The incidence of LBW (weigth < 2,500 g) was significantly higher among infants of mothers in the CWC/CQ group (23.9%) as compared with those of mothers in the IPTp/CQ (15.6%) and IPTp/SP (11.6%) groups (p = 0.02)

**Conclusion:**

Intermittent preventive treatment with SP has shown clear superiority in reducing adverse outcomes at delivery, as compared with intermittent preventive treatment with CQ and classical chemoprophylaxis with CQ.

## Background

In malaria endemic countries, pregnant women, along with children under five years, represent the most vulnerable group to *Plasmodium falciparum *infection [1,2]. Such infection often increases the risk of morbidity and mortality for the mother and her child. Indeed, in malaria stable transmission conditions, it has been shown that pregnancy associated with malaria increases the risk of maternal anaemia, stillbirths and low birth weight (LBW) [3,4]. Over 26% of anaemia in pregnancy is attributable to malaria, and malaria-related maternal deaths are reaching an unacceptable rate of 23%[5]. These adverse outcomes have been described as the consequences of placental sequestration of *P. falciparum*. In addition, because of this sequestration, peripheral blood film microscopy usually underestimates the prevalence of placental malaria [6,7].

In areas of high or moderate transmission, most malaria infections in pregnant women are asymptomatic and therefore will not be presented to any clinic for diagnosis and management. It is important that in such conditions, strategies to protect mothers during their pregnancy include the use of insecticide-treated material (ITM) as well as effective anti-malarial drugs used in intermittent preventive treatment (IPT) regimens and case management [8-10].

In Burkina Faso, until recently, chloroquine (CQ) was the only drug recommended by the National Malaria Control Programme for use in chemoprophylaxis against malaria in pregnant women. However, with the increasing resistance of *P. falciparum *to chloroquine and the evidence of the efficacy of IPT in pregnant women using sulphadoxine-pyrimethamine (SP), new guidelines were issued in February 2005 for a policy change regarding the prevention of malaria during pregnancy.

The aim of the present study was to provide data to the National Malarial Control Programme for an evidence-based policy change decision making process. The study has assessed the efficacy of three possible approaches being considered at the time of the study initiation. It compares the efficacy of two IPT regimens (using CQ or SP) with the classical chemoprophylaxis regimen using CQ in reducing the adverse outcomes of malaria infection for the mother and the foetus.

## Methods

### Study site and population

Details on the study site and methods were published by Ouedraogo *et al *[11]. Briefly, the study was conducted in the Antenatal Care Unit of the Health District of Bousse, a district located around 50 km north-west of Ouagadougou, the capital of Burkina Faso. The district covers a total population of 124,285 inhabitants. The majority of this population is farmers, illiterate, and belongs to the Mossi ethnic group.

The health district is situated within the Mossi Central Plateau, in the Sudano-Sahelian zone, with a tropical climate characterized by two seasons: the dry season (November - May), in which malaria transmission is very low, and the rainy season (June - October), in which malaria transmission is high. The annual rainfall is in the range of 600 to 900 mm. The main malaria vectors are *Anopheles gambiae, Anopheles arabiensis *and *Anopheles funestus*. Two chromosomal forms each of *An. gambiae *and *An. funestus *are sympatric in the study area. The annual entomological inoculation rates range from 10 to 500 infective bites per individual. *Plasmodium falciparum *is responsible for more than 90% of malaria infections.

In terms of infrastructure, the Health District includes the District Hospital, which serves as referral center for 11 community health clinics named Centre de Santé et de Promotion Sociale (CSPS). The district hospital comprises a medical unit, a surgery unit and the Child and Maternal Health Unit (CMHU). Antenatal care and family planning services are offered by the CMHU. Therefore, the study staff recruiting team was based there to screen and enrol women seeking antenatal care who fulfilled the study inclusion criteria. Among other criteria, eligible women were required to be permanent residents of the Health District area with a gestational age between 15 and 36 weeks at the time of the visit, and to have provided freely given written informed consent. All women who were otherwise eligible but who had a history of allergic reaction to any of the study drugs (CQ and SP) were excluded.

### Selection, randomization and treatment of the study participants

In Burkina, three antenatal care unit visits are recommended during pregnancy. The initial visit (ANC_1_) is scheduled between the 15^th ^and 25^th ^week of gestational age; the second visit (ANC_2_) is scheduled between the 28^th ^and 32^nd ^week of gestational age, and the third visit (ANC_3_) is scheduled between the 33^rd ^and the 36^th ^week of gestational age.

Pregnant women undergoing their ANC_1 _who were willing to participate in the study were assessed with regard to the inclusion and exclusion criteria. Eligible women were randomly assigned to one of the three treatment arms using a computer generated random list.

The study pharmacist, who did not take part in any other activities in the study, prepared an individual randomization code envelope from the randomization list. The sealed envelopes were sequentially numbered. When an eligible woman joined the study, the envelope with the lowest available number was opened, revealing the assignment of the participant to the appropriate treatment arm.

The first treatment arm was classical weekly chemoprophylaxis using chloroquine (CWC/CQ). Participants in this arm received, upon enrolment, a curative dose of CQ (600 mg/day at Day 0, then 300 mg/day at Day 1 and Day 2) followed by 300 mg/week until 6 weeks post delivery. The second and third treatment arms consisted of intermittent preventive treatment in pregnancy (IPTp) regimens. For the second arm (IPTp/CQ), the IPTp regimen was a complete cure with CQ at each of the antenatal care visits (ANC_1_, ANC_2 _and ANC_3_). A total dose of 600 mg/day of CQ was administered at Day 0, then 300 mg/day at Day 1 and Day 2. In the third arm (IPTp/SP), the IPTp regimen consisted of a single curative dose of 1.5 g/0.075 g sulphadoxine pyrimethamine at each of the antenatal care visits (ANC_1_, ANC_2 _and ANC_3_). The treatment administration was not supervised. Each woman was given medication, according to her treatment arm, for self-administration after leaving the clinic.

### Follow-up of the study participants

Enrolled pregnant women were followed up to delivery. At enrolment and subsequent visits, the study midwife collected the study data using a standardized questionnaire. During the pregnancy, the following data were collected: sociodemographic factors (age, village, school attendance, marital status), obstetrical past history (gravidity, parity), pregnancy related factors (number of ANC visits, oedema, malaria episode), and malaria prevention measures (chemoprophylaxis and use of bed nets). A physical examination was performed to measure axillary temperature, blood pressure, weight and uterine height.

A blood sample was obtained by fingerprick for a blood smear and haemoglobin (Hb) was measured using a portable hemocue (*HemoCue*^® ^AB, Ängelholm, Sweden). Anaemia was defined as Hb < 11 g/dL. An Hb level between 8 and 11 g/dL was considered mild to moderate anemia and an Hb level ≤ 8 g/dL as severe anaemia.

In participants diagnosed with fever (axillary T° ≥ 37.5°C), a malaria rapid diagnosis test (OPTimal^®^) was done to allow prompt management of the case. Results of the impact of the three treatment arms in terms of maternal infection and prevalence of maternal anaemia have been published elsewhere by Ouedraogo *et al *[11]

At delivery, data regarding newborn characteristics (vital status at birth, birth weight, sex and the presence of twins or malformation) were collected. Birth weight was measured using an electronic digital scale (± 10 grams) (Tanita Corporation, Tokyo, Japan). In addition, cord blood samples were obtained for smears and placental blood smears were obtained from the maternal side of the placenta. A malaria smear was also obtained from the mother by fingerprick. No sample was obtained from the neonate to check for malaria parasites.

### Management of anaemia and malaria cases

Study participants found to be anaemic at ANC visits (Hb level < 11 g/dL) were treated, based on national guidelines, with ferrous sulfate (200 mg) and folic acid (0.25 mg) given as a single combined tablet daily for 30 days. Confirmed cases of malaria (axillary T° ≥ 37.5°C and with a positive rapid diagnosis test) were given treatment with quinine, based on the national malaria treatment guidelines for pregnant women.

### Laboratory methods

All blood films (maternal, placental and cord) were stained with 10% Giemsa for 45 min, and read at the Centre National de Recherche et de Formation sur le Paludisme (CNRFP) immunoparasitology laboratory in Ouagadougou. For thick films, parasites and leukocytes were counted in the same fields until 500 leukocytes were counted. Parasite densities were estimated using an assumed leukocyte count of 8,000 leukocytes/μL. Thin films were then used to determine species when thick films were positive. All slides were double read by two independent microscopists. If the ratio of densities from the first two readings were > 1.5 or < 0.67 or if < 30 parasites were counted with a difference in the number of parasites > 10, the slide was evaluated a third time. The final result was the geometric mean of the parasite density of the two most concordant results of the three readings. When the discordance was only in terms of positivity, the slide was also evaluated a third time and the definitive result was based on the majority verdict for positivity. Placental histology was not performed.

### Statistical analysis

The data collected in the study questionnaire were verified then double entered using Epi-info 6.04 fr (CDC, Atlanta, USA). Data were analysed using Epi-info and Sata 7.0. Analysis was by intent-to-treat. The analysis included data from births of all enrolled participants who had taken the study treatment at least once and who delivered at the study centre clinic for which data are available. Continuous normally distributed data were described by the mean and standard deviation, and non-normally distributed data by the median or geometrical mean and range.

Proportions were compared using the Chi square test and normally distributed continuous variables were compared using Student's t test. Statistical tests were considered significant when the two-sided P value was < 0.05.

### Ethical considerations

The study was initially discussed with health authorities and local leaders to obtain their assent. The study was submitted to and approved by the National Ethical Review Committee in Burkina Faso and the Ethical Committee of WHO/TDR. A written informed consent was obtained from all the women prior to their enrolment in the study. For illiterate mothers, the informed consent discussion process was witnessed by an impartial individual.

## Results

### Characteristics of the study participants at enrolment

**Figure 1:**
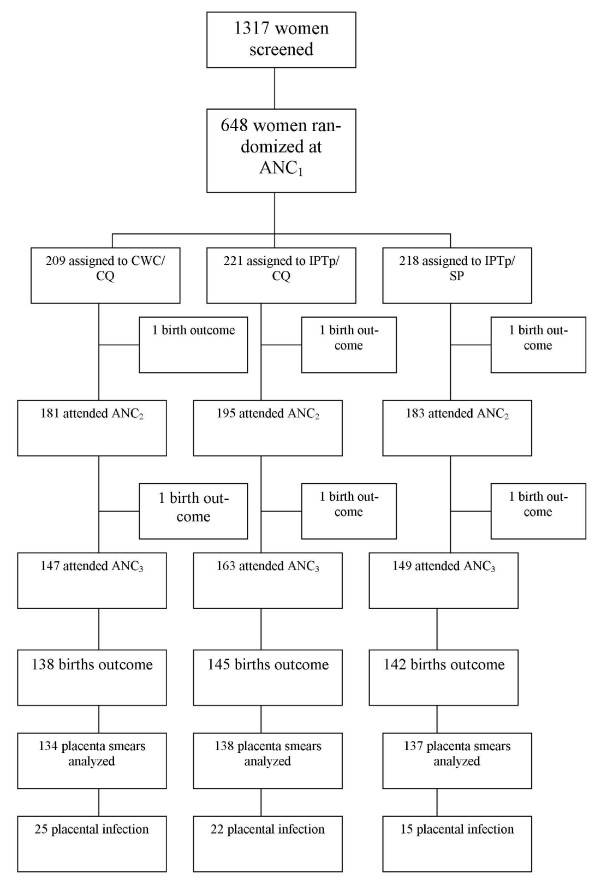
**The trial profile**.

The trial profile is provided in Figure 1. In total, 648 pregnant women were recruited and randomized to one of the three study arms. Baseline characteristics were similar within the study groups (Table 1). The mean age (SD) of the participants was 23.7 (5.9) and 39.7% of them (257) were primigravidae. The primigravidae and secundigravidae represent 58.7% of the total sample size. Over half of the women (50.8%) were parasitaemic at enrolment; this proportion was significantly higher for women randomized to the IPTp/SP group (p = 0.005). Overall, the geometric mean of the parasite density was 855.4 (95% ci 691.6-1058.06). There was no difference between treatment groups. The rate of use of insecticide impregnated materials (bed nets and/or curtains) was very low in the study population and was comparable across the study groups.

**Table 1:** Baseline characteristics of the study participants

**Characteristics**	**CWC/CQ** **n = 209**	**IPTp/CQ** **N = 221**	**IPTp/SP** **n = 218**	**All participants N = 648**	**P**
Age (mean ± SD; range)	24.3 ± 6.3 (16; 51)	23.3 ± 5.7 (17; 42)	23.6 ± 5.8 (17; 45)	23.7 ± 5.9 (16; 51)	0.16
Parity (mean ± SD; range)	1.7 ± 0.01 (0; 8)	1.5 ± 0.01 (0; 9)	1.4 ± 0.01 (0; 7)	1.5 ± 0.01 (0; 9)	0.07
Primigravidae (%)	32.2	42.5	44.0	39.7	0.02
Secundigravidae (%)	21.2	19.0	17.0	19.0	0.56
Multigravidae (%)	46.6	38.5	39.0	41.3	0.18
Weight (mean ± SD; range)	54.6 ± 6.3 (41, 80)	54.8 ± 8.3 (39; 79)	55.7 ± 8.2 (40;105)	55.2 ± 7.7 (39;105)	0.54
Gestational age (mean ± SD)	21.8 ± 2.8	21.6 ± 2.8	21.75 ± 2.7	21.75 ± 2.7	0.67
Uterine fundal Height (mean ± SD; range)	21.4 ± 3.3 (13; 30)	21.2 ± 3.2 (13; 30)	21.2 ± 2.8 (14; 27)	21.3 ± 3.1 (13; 30)	0.66
Parasitaemia (mean, 95% CI)	666.6 (450.9 985.6	908.1 (636.5-1295.8)	951.8 (661.5-1369.4)	855.4 (691.6-1058.06)	0.27
Parasitaemia (%)	41.5%	55.0%	55.3%	50.8%	0.005
Haemoglobin level (mean ± DS; range)	10.9 ± 1.5 (5.1;15.2)	10.9 ± 1.4 (7.0; 14.0)	10.4 ± 1.3 (7.8; 14.6)	11.0 ± 1.4 (5.1; 15.2)	0.81
Impregnated bed nets (%)	4.3	6.8	5.0	5.4	0.50
Non-impregnated bed nets (%)	9.6	6.3	6.9	7.56	0.39
Impregnated curtains (%)	2.9	2.3	2.3	2.5	0.89
Non-impregnated curtains (%)	56.3	67.0	59.6	61.0	0.06

One woman (0.24%) in the IPTp/SP group died during the delivery as the consequence of eclampsia. Other complications during the delivery included peripartum haemorrhage (1.18%), placental retention (1.9%), and retro-placental haemorrhage (1.4%). Two stillbirths (0.4%) were recorded.

### Placental malaria and low birth weight

A total of 423 women were followed up until delivery; they were similarly distributed in the three treatment groups (see Table 2). Overall, peripheral maternal *P. falciparum *infection at delivery was found in 25.8% of the women. The proportion of women with maternal infection was significantly lower in the IPTp/SP group than in the CQ group (P << 0.000). Among all the delivering primigravidae, the proportion of women with maternal infection was almost double in the CWC/CQ group compared with the IPT groups. The difference was statistically significant (p << 0.000). Among the secundigravidae, although the highest proportion of infected mothers was observed in the IPT groups (47.8% for the IPTp/CQ group and 22.7% for the IPTp/SP group) as compared to the CWC/CQ group (20.0%), this result did not reach significance level (p = 0.07).

**Table 2:** Outcomes at delivery

**Characteristics**	**CWC/CQ** **(n = 137)**	**IPTp/CQ** **(n = 145)**	**IPTp/SP** **(n = 141)**	**All** **(N = 423)**	**P**
Maternal parasitaemia					
*Overall*	32.8	30.9	13.9	25.8	0.00
*Primigravidae*	57.8	36.4	16.9	35.2	0.00
*Secundigravidae*	20.0	47.8	22.7	30.0	0.07
*Multigravidae*	19.7	19.7	7.1	15.7	0.10
Placental malaria (%)					
*Overall*	18.8	15.9	10.6	15.6	0.14
*Primigravidae*	31.1	25.4	15.9	23.4	0.16
*Secundigravidae*	17.9	20.0	13.6	17.3	0.84
*Multigravidae*	10.8	4.9	3.5	6.6	0.22
Placental malaria Parasite densities (mean* 95%CI)	666.7 (244.8-1815.75)	1535.8 (560.8-4205.9)	1030.1 (260.8-4069.0)	996.5 (544.9-1822.3)	0.53
Cord blood parasitaemia (%)					
*Overall*	2.9	0.7	0.7	1.4	0.19
*Primigravidae*	2.2	1.7	1.6	1.8	0.97
*Secundigravidae*	7.14	0	0	2.7	0.18
*Multigravidae*	1.54	0	0	0.6	0.40
Birth weight (mean, range)	2749 (1100-3900)	2819 (1500-4100)	2836 (1500-4000)	2802 (1100-4100)	0.15
Low birth weight** (%)					
*Overall*	23.9	15.6	11.4	16.9	0.02
*Primigravidae*	40.9	18.9	14.5	23.2	0.004
*Secundigravidae*	18.5	16.00	14.3	16.4	0.92
*Multigravidae*	16.9	13.6	8.9	13.3	0.43
Premature delivery	28.3%	26.2%	30.3%	28.2%	0.75
Spontaneous abortion	1.5%	1.4%	0.7%	1.2%	0.81

* Geometric mean

** CWC/CQ = 134; IPTp/CQ = 138; IPTp/SP = 137

The prevalence of placental malaria was 18.8% in the CWC/CQ group; 15.9% in the IPTp/CQ group and 10.6% in the IPTp/SP group. These trends remained unchanged after stratification according to level of gravidity (Table 2). Statistically significant differences were only found between the CWC/CQ group and the IPTp/SP group (p = 0.049).

After adjustment, women in the IPTp/SP group had a lower risk of placental malaria when compared with those in the CWC/CQ group (OR = 0.44 (95% ci 0.22-0.90), p = 0.025). No significant difference was observed between the women in the IPTp/CQ group and those in the CWC/CQ group (OR = 0.75 (95% ci 0.4-1.41), p = 0.39). The mean birth weight was 2,802 g (1,100 to 4,100); there were no differences among the treatment groups in mean birth weight.

In total, 17.3% of the newborns had a low birth weight (LBW) (weight < 2,500 g). The incidence of LBW was significantly higher among infants of mothers in the CWC/CQ group (23.9%) as compared with those of mothers in the IPTp/CQ (15.6%) and IPTp/SP (11.6%) groups (p = 0.02). The use of IPTp either with CQ or SP was associated with lower risk of LBW; thus, the adjusted risk of LBW was significantly lower for infants of mothers in the IPTp/SP group as compared with those from the CWC/CQ group (OR = 0.38 (95% ci (0.19-0.72), p = 0.004). A similar situation was observed when comparing infants of mothers in the IPTp/CQ and those of mothers in the CWC/CQ group (OR = 0.53, 95% ci (0.29-0.98), p = 0.045).

As presented in Table 2, among primigravidae, the proportion of infants with LBW was significantly higher in the CWC/CQ group (40.9%, versus 18.9% in IPTp/CQ and 14.5% in IPTp/SP groups, p = 0.004). In secundigravidae and multigravidae mothers, the proportion of infants with LBW was also higher in the CWC/CQ group, but this difference did not reach statistical significance.

Cord blood parasitaemia was found in six cases; four of them were from the CWC/CQ group, one in the IPTp/CQ group and one in the IPTp/SP group. The difference was not statistically significant (p = 0.19).

## Discussion

This study compared the efficacy of three treatment regimens for the prevention of placental malaria: classical chemoprophylaxis using CQ, intermittent preventive treatment using CQ, and intermittent preventive treatment using two doses of SP.

This study was conducted at the time of a transition period in Burkina Faso; CWC/CQ was still the regimen recommended by the National Malaria Control Programme for preventing malaria during pregnancy, but the declining efficacy of CQ had lead to the initiation of discussion of a change in this policy.

The IPTp/SP regimen was more effective than the other two regimens in preventing placental malaria. No difference was found between the IPTp/CQ and the CWC/CQ regimens. Significant differences were only found between the CWC/CQ and IPTp/SP groups, suggesting that, in addition to the high resistance of *P. falciparum *to CQ in the area, [12,13] the low efficacy observed with the CWC/CQ is probably also related to the issue of compliance. Compliance issues with this type of treatment regimen have been commonly observed in women living in settings comparable to those of our study population.

The risk of placental malaria was significantly higher in the CWC/CQ as compared to the IPTp/SP group. This finding is consistent with those reported in similar studies which have shown that IPTp with SP is more efficacious than chemoprophylaxis with CQ in preventing placental malaria [14-16].

The IPTp/SP regimen was also more efficacious in reducing the proportion of LBW as compared with the two other CQ regimens. These findings are consistent with what has been reported in similar studies [14,17]. The efficacy of all three-treatment regimens in preventing maternal infection, placental malaria, and low birth weight increased with gravidity. This finding confirms the high burden of malaria for women with low gravidity in areas with intense parasite transmission. Indeed, in high malaria endemicity settings, primigravidae and, to a lesser extent, secundigravidae are known to be more highly affected than other parities [18].

The proportion of premature delivery was unexpectedly high in all three-treatment regimens. No significant differences were observed between the groups. Considering the efficacy of IPTp/SP in preventing placental malaria infection, and CQ's potential antipyretic effect (mediated by inhibition of tumour necrosis factor) [19], one may expect lower premature delivery rates comparable with those reported in other studies using the same anti-malarial drugs [14,15,20]. It is possible that in this case, the gestational ages were overestimated in some cases due to a recall bias (of the date of last menstrual period, which is used to estimate the gestational age at the first antenatal clinic visit) by the mothers.

## Conclusion

From the perspective of reducing the adverse consequences of malaria on pregnancy outcomes, IPTp/SP has shown clear superiority as compared with IPTp/CQ and classical chemoprophylaxis with CQ. These results indicate that IPTp/SP treatment could be adopted as the official recommended treatment for pregnant women, while alternative drugs for IPT are being investigated.

## Competing interests

The authors declare that they have no competing interests.

## Authors' contributions

SBS conceived the study and its design; he coordinated the data collection, the analysis and interpretation of the results and the review of the manuscript. ABT participated to the design of the study, the data collection and analysis and has drafted the manuscript. AO, ECB, AD, ATK and IN participated in the data collection and data analysis and interpretation. All authors have read and approved the final manuscript.
